# Seasonal Variation in Essential Oil Composition and Antioxidant Capacity of *Aniba canelilla* (Lauraceae): A Reliable Source of 1-Nitro-2-phenylethane

**DOI:** 10.3390/molecules28227573

**Published:** 2023-11-14

**Authors:** Ellen de Nazaré S. da Cruz, Luana de Sousa P. Barros, Bruna de A. Guimarães, Rosa Helena V. Mourão, José Guilherme S. Maia, William N. Setzer, Joyce Kelly do R. da Silva, Pablo Luis B. Figueiredo

**Affiliations:** 1 Programa Institucional de Bolsas de Iniciação Científica, Universidade Federal do Pará, Belem 66075-900, Brazil; ellen.cruz@icen.ufpa.br; 2 Laboratório de Química dos Produtos Naturais, Centro de Ciências Biológicas e da Saúde, Universidade do Estado do Pará, Belem 66087-662, Brazilbruna.guimaraes@aluno.uepa.br (B.d.A.G.); 3 Programa de Pós-Graduação em Ciências Farmacêuticas, Instituto de Ciências da Saúde, Universidade Federal do Pará, Belem 66075-900, Brazil; gmaia@ufpa.br; 4 Programa de Pós-Graduação em Química, Universidade Federal do Pará, Belem 66075-900, Brazil; 5 Laboratório de Bioprospecção e Biologia Experimental, Universidade Federal do Oeste do Pará, Santarem 68035-110, Brazil; 6 Aromatic Plant Research Center, 230 N 1200 E, Suite 100, Lehi, UT 84043, USA; wsetzer@chemistry.uah.edu; 7 Programa de Pós-Graduação em Biotecnologia, Universidade Federal do Pará, Belem 66075-900, Brazil

**Keywords:** benzenoids, precious bark, volatiles, *Aniba canelilla*, hydrodistillation, antioxidant capacity

## Abstract

*Aniba canelilla* (Kunth) Mez essential oil has many biological activities due to its main compound 1-nitro-2-phenylethane (1N2F), followed by methyleugenol, a carcinogenic agent. This study analyzed the influence of seasonality on yields, antioxidant capacity, and 1N2F content of *A. canelilla* leaf and twig essential oils. Essential oils (EOs) were extracted with hydrodistillation and analyzed with gas chromatography coupled to mass spectrometry and a flame ionization detector. Antioxidant capacity was measured using the free radical scavenging method (DPPH). Chemometric analyses were carried out to verify the influence of climatic factors on the production and composition of EOs. 1-Nitro-2-phenylethane was the major constituent in *A. canelilla* EOs throughout the seasonal period (68.0–89.9%); methyleugenol was not detected. Essential oil yields and the 1N2F average did not show a statistically significant difference between the dry and rainy seasons in leaves and twigs. Moderate and significant correlations between major compounds and climate factor were observed. The twig oils (36.0 ± 5.9%) a showed greater antioxidant capacity than the leaf oils (20.4 ± 5.0%). The PCA and HCA analyses showed no statistical differences between the oil samples from the dry and rainy seasons. The absence of methyleugenolin in all months of study, described for the first time, makes this specimen a reliable source of 1N2F.

## 1. Introduction

The Lauraceae comprises around 50 genera and 2500 to 3000 species distributed in tropical and subtropical regions; mainly, taxa are aromatic trees and shrubs rich in essential oils [1,2]. Lauraceae is native and non-endemic in Brazil, with 27 genera and 466 species of trees, shrubs, and lianas, known as climbers [3].

The Lauraceae has economic potential in several industrial sectors, such as food, wood, pharmaceuticals, and perfumery. Regarding its ethnobotany, its taxa are used to treat several pathologies [4,5]. Among the genera of this family, *Aniba* species have many scientific studies highlighting their pharmacological potential, emphasizing *A. rosaeodora* Ducke, *A. parviflora* (Meisn.) Mez, and *A. canelilla* (Kunth) Mez [6,7].

*Aniba canelilla* (Kunth) Mez is an aromatic species popularly known as “precious bark”, “false cinnamon”, “canelão”, and “precious leaf”. It is native and endemic to Brazil and found in the north, central-west, and southeast regions of the country. It is widely used in popular medicine to treat inflammation, intestinal pain, respiratory diseases, microbial, and parasitic infections [3,8]. Furthermore, *A. canelilla* essential oil is a natural antioxidant for food preservation and disease control, presenting high potential for use in cosmetics and pharmaceutical products [8].

In the aromatic and medicinal plants market, essential oils (EOs) are widely sought after due to their applications in perfumery, beverages, food, and cooking. EOs can be present in different parts of plants, such as leaves, seeds, stems, bark, and roots [9]. Moreover, they are used in traditional medicine as antimicrobial agents, as they are biologically active compounds with important health effects [10,11].

*A. canelilla* essential oil has antioxidant, antinociceptive, anti-inflammatory, anxiolytic, anticholinesterase, fungicidal, trypanocidal, leishmanicidal, cardiomoderating, and hypotensive properties [8]. The odorous principle of *A. canelilla* leaves, bark, and wood comes from the compound 1-nitro-2-phenylethane. This constituent is volatile, with an aroma similar to cinnamon, and stands out for its anti-inflammatory, antinociceptive, and vasorelaxant potential [12,13,14].

Nitro-substituted compounds have demonstrated broad biological activities and their pharmacological potential has been reviewed [15]. Furthermore, the presence of the hydrophobic phenyl group makes 1N2F lipophilic and affects its membrane and blood–brain barrier transport ability [16,17].

On the other hand, there are reports in the literature indicating the presence of methyleugenol in the *A. canelilla* essential oil, which is considered a carcinogen and mutagen with a solid link to safrole [18,19]. Due to the biological activities of this species and its possible industrial and pharmacological applications, the objective of this study was to evaluate its antioxidant potential and the influence of climatic factors on the yields and 1-nitro-2-phenylethane contents of the essential oil of *A. canelilla*.

## 2. Results and Discussion

### 2.1. Essential Oil Yields vs. Environmental Conditions

**Figure 1:**
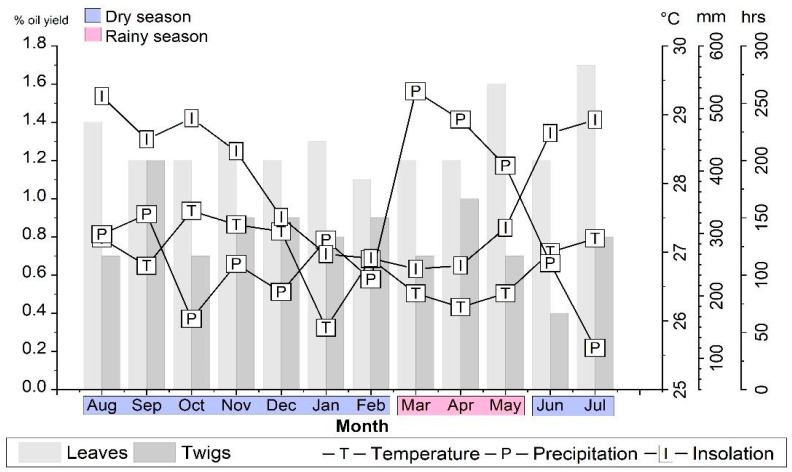
Relationship between climatic parameters and essential oil yields of leaves and twigs of *Aniba canelilla* in this seasonal study.

The climatic parameters (precipitation, temperature, and insolation) were monitored from August 2021 to July 2022 to evaluate the influence of seasonality on the composition and yields of *A. canelilla* essential oil. The average precipitation values ranged from 116.6 mm (July) to 527.4 mm (March), the average temperature from 25.9 °C (January) to 27.6 °C (October), and the values of insolation from 105.4 h (March) to 256.1 h (August). Based on rainfall data, the months of March to May comprise the rainy season with an average rainfall of 472.5 ± 60.2 mm, and the months of August to February, in addition to June to July, comprise the dry season with an average rainfall of 237.2 ± 67.8 mm (see Figure 1). In the seasonal study of *Lippia alba* (Mill.) N.E.Br. ex Britton & P. Wilson (Verbenaceae), the dry season also comprised the months of August to February and the rainy period from March to May [20].

The *A. canelilla* specimen was collected in the city of Belém, located in northern Brazil, which has a predominantly hot and humid climate. The climate of the Amazon region has only two delimited seasons, the rainy and the dry. Despite this, the seasons can change according to the atmospheric phenomena in the region [21].

In this seasonal study, the oil yields of *A. canelilla* leaves ranged from 1.1% (February) to 1.7% (July), and of the twigs ranged from 0.4% (June) to 1.2% (September). The essential oil yields of *A. canelilla* leaves (1.1–1.7%; 1.3 ± 0.2) were higher than the twigs (0.4–1.2%; 0.8 ± 0.2) in all months of this study, except in September, where they were the same (1.2%). Furthermore, the average yield of leaves (1.3 ± 0.2) and twigs (0.8 ± 0.2) showed a statistical difference (*p* < 0.05) in the Tukey test.

A specimen of *A*. *canelilla* collected in Belém, Pará, Brazil, presented an oil yield of 1.5% for leaves and 1.0% for twigs [6]. Another specimen collected in Amazonas also showed higher oil yields in the leaves (1.3%) than in the twigs (1.2%) [22].

The essential oil yields of leaves (L) and twigs (T) did not show a statistically significant difference between the dry (L: 1.3 ± 0.2; T: 0.8 ± 0.2) and rainy (L: 1.3 ± 0.3; T: 0.8 ± 0.2) seasons. In this sense, the influence of seasonality on the EOs of a specimen of *Psidium acutangulum* DC. (Myrtaceae) collected in the city of Belém, Pará, Brazil, has been reported; the oil yields also showed no statistical differences between the dry (0.7 ± 0.3%) and rainy (0.9 ± 0.2%) periods [23]. Regarding climatic factors vs. essential oil yields, the Pearson correlation coefficient (r) analysis showed that there was no significant correlation between the yields of *A. canelilla* leaves and twigs, respectively, with regard to temperature (r = 0.01 and r = −0.11), insolation (r = 0.30 and r = −0.20), or precipitation (r = −0.17; r = 0.10), as shown in Table 1.

Yields and composition of secondary metabolites can be affected from plant formation to final isolation [24]. For example, the EO present in the leaves of *Nectandra grandiflora* Nees (Lauraceae), collected in the Rio Grande do Sul (Brazil), showed seasonal variability, with the highest yield in spring (0.75 ± 0.06%) and the lowest yield in the winter (0.39 ± 0.02%) [25]. Moreover, *Ocotea porosa* (Nees & Mart.) had an oil content of 0.82%, while *Ocotea quixos* (Lam) Kosterm had an EO content equivalent to 1.6% [25].

### 2.2. Chemical Composition vs. Environmental Conditions

GC-MS and GC-FID identified and quantified the oil constituents from the leaves and twigs of *A. canelilla* during the twelve months of this study (August 2021 to July 2022). In total, 61 volatile compounds were identified, representing an average of 98.3% of the total composition of the oils (Table 2 and Table A1). The predominant class of EOs in the leaves (L) and twigs (T) were benzenoids (L: 70.8–87.5%; T: 72.5–91.0%), followed by oxygenated monoterpenoids (L: 2.3–4.5%; T: 5.3–21.8%), sesquiterpene hydrocarbons (L: 1.1–10.5%; T: 0.1–0.5%), oxygenated sesquiterpenoids (L: 1.4–9.1%; T: 0.9–3.9%), and monoterpene hydrocarbons (L: 0.1–4.8%; T: 0.1–5.9%).

The main compound of the EOs was 1-nitro-2-phenylethane (1N2F) in the leaves (68.0–85.2%; 78.7 ± 5.5%) and twigs (71.3–90.0%; 80.7 ± 6.6%). However, unlike the oil yields, there was no statistically significant difference in the Tukey test (*p* > 0.05) between the amounts of 1N2F in the leaves and twigs of *A. canelilla*.

The 1N2F content ranged from 68.0% (February) to 85.2% (March) in the leaves and 71.3% (December) to 89.9% (March) in the twigs. The average amounts of 1N2F in the leaves and twigs of *A. canelilla* were higher in the rainy season (F: 84.5 ± 1.2; T: 87.8 ± 2.1) than in the dry season (L: 76.8 ± 5.0; T: 78.3 ± 5.8). The average concentration of 1N2F in the leaves (76.9 ± 4.1%) and twigs (71.3–81.7%) did not show a statistical difference (*p* < 0.05) in the Tukey test. Furthermore, the average contents of 1N2F did not show a statistically significant difference between the dry (L: 76.8 ± 5.0; T: 78.3 ± 5.8) and rainy (L: 84.5 ± 1.2; T: 87.8 ± 2.1) seasons in the leaves and twigs.

**Figure 2:**
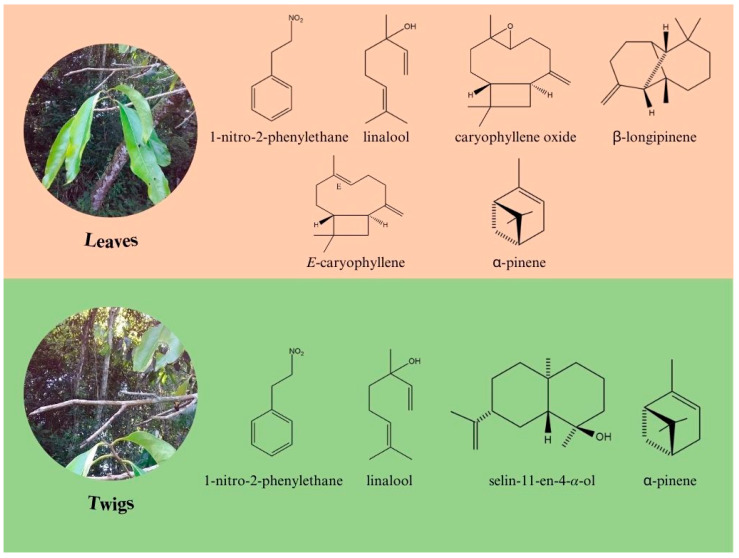
Chemical structures of the main compounds identified in the essential oils of *A. canelilla* leaves and twigs.

Other constituents were also identified in *A. canelilla* EOs leaves and twigs, such as the monoterpene hydrocarbon α-pinene (L: 0.0–2.2%; 0.5 ± 0.6%; T: 0.0–2.2%; 0.7 ± 0.8%) and the oxygenated monoterpenoid linalool (L: 1.9–3.5%; 2.7 ± 0.5%; T: 4.5–20.1%; 11.3 ± 4.7%), the sesquiterpene hydrocarbons *E*-caryophyllene (L: 0.2–6.6%; 2.5 ± 2.3%; T: 0.1–0.3%; 0.2 ± 0.1%) and β-longipinene (L: 0.0–4.8%; 1.4 ± 1.4%; T: < 0.1%), and the oxygenated sesquiterpenoids selin-11-en-4α-ol (L: 0.0–1.1%; 0.5 ± 0.5%; T: 0.0–2.5%; 1.2 ± 1.0%) and caryophyllene oxide (L: 0.7–5.6%; 4.5 ± 1.4%; T: 0.0–0.4%; 0.2 ± 0.1%). The chemical structures of these compounds are shown in Figure 2.

The 1N2F (L: 71.2%; T: 68.2%) [14] and (L: 88.3%; T: 70.9%) [6] was previously identified in high amounts in *A. canelilla* EOs. Both studies found that the levels of 1N2F in the leaves were higher than in the twigs, which vary from the results of this study.

Based on the analysis of the Pearson correlation coefficient (r) shown in Table 1, there was a moderate and significant positive correlation (*p* < 0.05) between precipitation and the 1N2F contents in the leaves (r = 0.61) and twigs (r = 0.60), and a moderate negative correlation between 1N2F and temperature (r = −0.59) in the leaves. The twigs had a weak negative correlation between 1N2F content and temperature (r = −0.47) and insolation (r = −0.37).

Among the other chemical constituents present in *A. canelilla* EOs, those that significantly correlated with climatic parameters were linalool with precipitation in the twigs (r = −0.65), β-longipinene with insolation (r = 0.68) and precipitation (r = −0.64) in the leaves, selin-11-en-4α-ol with temperature (r = −0.68), insolation (r = −0.55), and precipitation (r = 0.72) in the twigs, and α-pinene with temperature (0.65) and insolation (r = 0.67). The sesquiterpenes *E*-caryophyllene and caryophyllene oxide did not significantly correlate with the climatic factors.

According to the statistically significant compounds classes, monoterpene hydrocarbons showed a moderate positive correlation with temperature (r = 0.69) and a strong positive correlation with insolation (r = 0.71) in the twigs. Oxygenated monoterpenoids showed a strong positive correlation with temperature (r = 0.78) in the leaves and a moderate negative correlation with precipitation in the leaves (r = −0.60) and twigs (r = −0.63). Sesquiterpene hydrocarbons showed a strong positive correlation with temperature (r = 0.70) and a strong negative correlation with precipitation (r = −0.70) in the leaves. Furthermore, oxygenated sesquiterpenoids showed a strong negative correlation with the average temperature (r = −0.70) in the twigs, and benzenoids showed a moderate negative correlation with temperature (r = −0.58) and a moderate positive correlation with precipitation in the leaves (r = 0.58) and twigs (r = 0.61).

**Figure 3:**
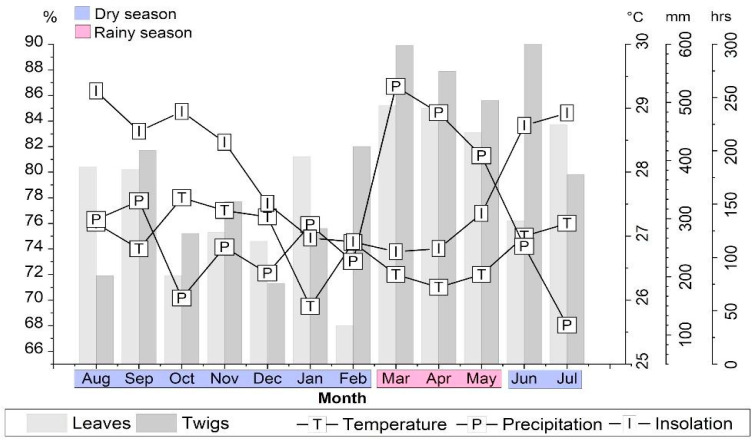
Seasonal study of 1-nitro-2-phenylethane in the leaves and twigs of *A. canelilla*.

Moreover, the highest amounts of 1N2F were obtained in March (F: 85.2%; T: 89.9%), a month with the highest precipitation (527.4 mm) and lowest sunshine (105.4 h), according to Figure 3.

The only seasonal study of *A. canelilla* reported in the literature indicated the presence of methyleugenol in its essential oil, which was used in foods as a flavoring agent. However, nowadays, methyleugenol is considered a carcinogen and mutagen with a strong link to safrol [18,19].

1N2F and methyleugenol contents varied with the season in a specimen from Carajás, southeast of Pará State [19]. During the rainy season, 1N2F showed higher amounts (95.3%) than methyleugenol (17.7%). Therefore, in the dry season, methyleugenol presented higher concentrations (45.8%) than 1N2F (39.0%). Comparing these results with the sample of *A. canelilla* collected in the city of Belém, state of Pará, the specimen of this article can be considered a natural and secure source of 1N2F.

Furthermore, the specimen in this study was evaluated with an in vivo experiment, where 1N2F increased antioxidant capacity and glutathione (GSH) concentrations, and reduced lipid peroxidation (both peritoneal and plasma). The essential oil decreased leukocyte migration induced by carrageenan, confirming its potential to treat inflammatory diseases and oxidative stress [28].

The volatile constituents of EOs are produced by secretory cells that minimize the risk of autotoxicity and allow the presence of high concentrations of secondary metabolites in places where their defense function may be vital [24]. Furthermore, several factors can lead to variations in the composition of secondary metabolites. Among these factors, seasonality stands out, a term used to designate variations that occur due to different times of the year [29].

Talking about the seasonal variation of *Aniba* species, the main chemical constituents identified in the essential oils of *A. parviflora* (Meisn) Mez. leaves were the monoterpenes: linalool, with variations from 14.07% (September) to 28.42% (March); α-phellandrene 5.66% (September) to 14.87% (March); *p*-cymene 2.74% (September) to 17.54% (March); and the oxygenated sesquiterpene spathulenol from 3.79% (December) to 7.0% (September) [30]. Thus, these findings show a great seasonal and intraspecific variation in the *Aniba* species.

### 2.3. Antioxidant Capacity vs. Environmental Conditions

#### DPPH Radical Scavenging

**Figure 4:**
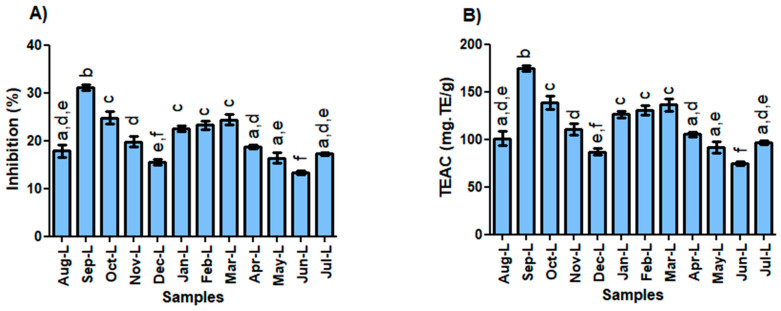

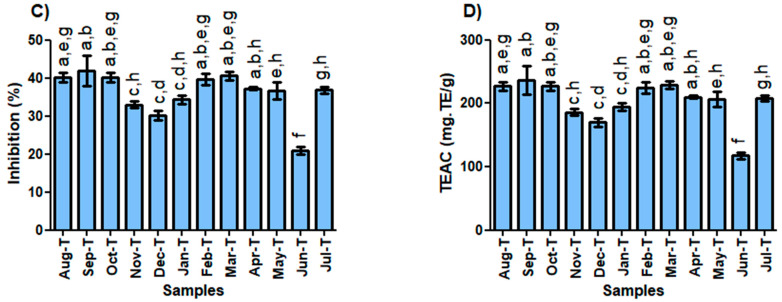
DPPH radical scavenging of the monthly oils of *Aniba canelilla*. (**A**) Inhibition of leaf oils; (**B**) TEAC of leaf oils; (**C**) inhibition of twig oils; (**D**) TEAC of twig oils. Values with the same letters in same graphic do not differ statistically in the Tukey test (*p* > 0.05).

The *A. canelilla* oils, obtained from a twelve-month collection process of leaves and twigs samples, showed a DPPH radical scavenging capacity with an average of 20.4 ± 5.0% for the leaf oils and 36.0 ± 5.9% for the twigs, as shown in Table A2 and Figure 4. The reaction kinetics were considered slow, with an average of 120 min. The highest percentage of inhibition of the DPPH radical was observed for the twig oils collected in September (42.0 ± 1.3%), March (40.6 ± 1.0%), August and October (40.2 ± 1.3%), February (39.8 ± 1.5), and April (37.2 ± 0.4). The total antioxidant capacity was expressed in values equivalent to the standard Trolox. TEAC (mg.TE/g) of the leaf oils showed an average of 114.4 ± 27.7, which is about ten times as low as Trolox; however, the TEAC for the twig oils showed an average of 203.0 ± 33.3, which is five times as low as Trolox. TEAC of the leaves and twigs were statistically different in the Tukey test (*p* < 0.05).

Based on Pearson’s correlation coefficient ^®^ analysis, the antioxidant activity of leaves showed no significant correlations with the major contents—1N2F (r = −0.197), linalool (r = 0.200), and caryophyllene oxide (r = −0.084)—or with the climatic parameters—insolation (r = −0.073), temperature (r = −0.127), rainfall (r = 0.159), and humidity (r = 0.206). Also, the antioxidant activity of twigs showed no significant correlations with the major contents—1N2F (r = −0.185), linalool (r = 0.103), and caryophyllene oxide (r = −0.336)—or with the climatic parameters—insolation (r = −0.093), temperature (r = −0.098), rainfall (r = 0.242), and humidity (r = 0.175).

A study of *Aniba canelilla* essential oils (110 to 1400 µg mL^−1^), obtained from Amazonas and Pará state (northern Brazil) and using Trolox as the standard, demonstrated a DPPH inhibition of 32.4 to 93.0%. For the methanolic extract (2 to 10 µg mL^−1^), the values ranged from 29.8 to 92.6%. They also reported the antioxidant capacity of 1N2F (200 to 1000 µg mL^−1^) and Trolox (2 to 10 µg mL^−1^); the values ranged from 11.5 to 63.2% and 21.5 to 96.7%, respectively [22]. In addition, the ethanolic extract of *A. canelilla* bark obtained from Pará state displayed optimum antioxidant activity (IC_50_ 1.80 ± 0.16). The same study demonstrated equivalence between the extract of *A. canelilla* and L-ascorbic acid. Its antioxidant potential was attributed to the presence of phenolic compounds, capable of interrupting the chain reactions caused by free radicals due to its ability to donate hydrogen atoms [31]. 

### 2.4. Multivariate Analysis of A. canelilla Leaf and Twig Essential Oils

**Figure 5:**
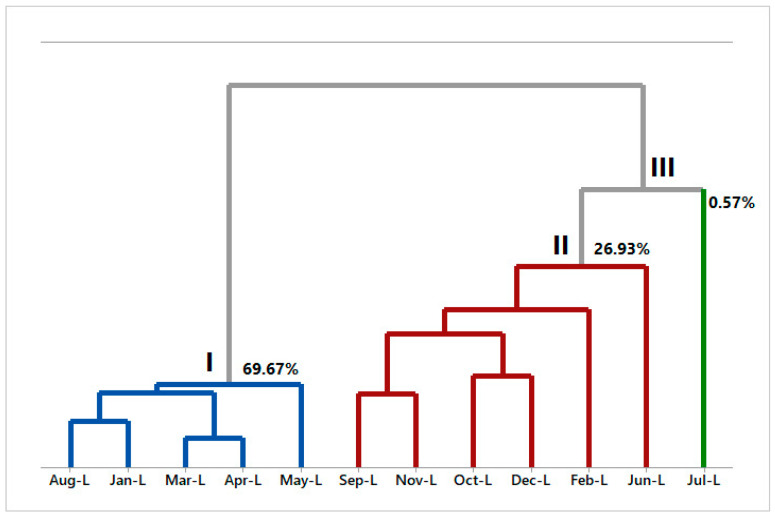
HCA analysis of the main compounds of essential oils from *A. canelilla* leaves.

Hierarchical cluster analysis (HCA) and principal component analysis (PCA) were performed using constituents with amounts above 2% in the EOs. The HCA and PCA plots were made separately for the leaves and twigs of *A. canelilla*. By applying hierarchical cluster analysis (HCA), it was possible to obtain the dendrogram that shows the three groups formed with no similarity from *A. canelilla* leaf volatiles (see Figure 5).

Group I includes the months of August, January, March, April, and May. Group II presents September, November, October, December, February, and June. On the other hand, group III only covers July.

**Figure 6:**
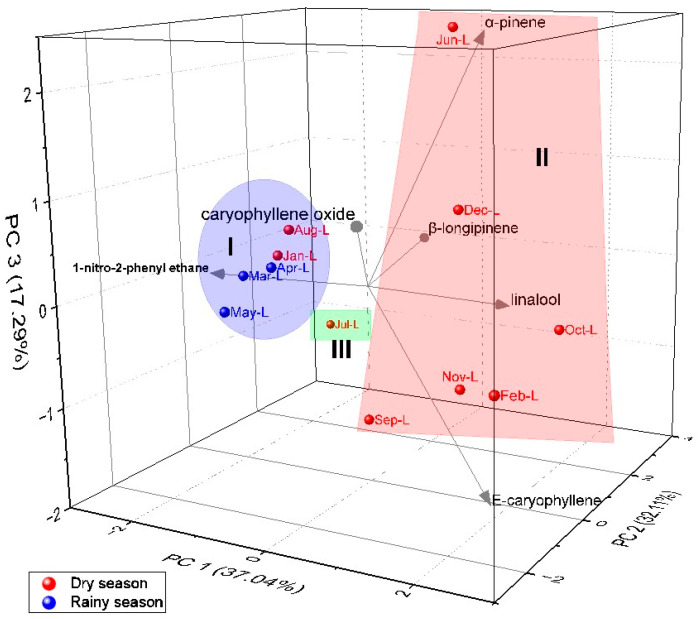
PCA analysis of the main compounds of essential oils from *A. canelilla* leaves.

The principal component analysis (PCA, Figure 6) elucidated 86.44% of the data variability. PC1 explained 37.04% of the data and presented negative correlations with 1N2F (r = −2.31) and β-longipinene (r = −0.49), and presented positive correlations with α-pinene (r = 1.24), linalool (r = 1.28), *E*-caryophyllene (r = 1.80), and caryophyllene oxide (r = 1.09). The second component (PC2) explained 32.11% of variability and showed negative correlations with 1N2F (r = −0.32), *E*-caryophyllene (r = −0.13), and caryophyllene oxide (r = −2.50), and positive correlations with α-pinene (r = 1.07), linalool (r = 1.88), and β-longipinene (r = 3.07). The third component (PC3) explained 17.29% of the data, presenting negative correlations with linalool (r = −0.37) and *E*-caryophyllene (r = −1.99), and positive correlations with α-pinene (r = 2.44), β-longipinene (r = 0.10), and caryophyllene oxide (r = 1.00). In relation to the HCA, the PCA analysis confirmed the formation of three distinct groups.

**Figure 7:**
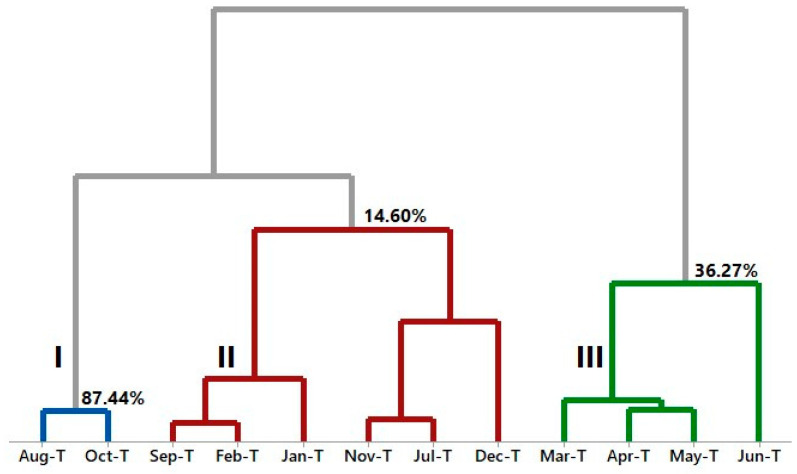
HCA analysis of the main compounds of essential oils from *A. canelilla* twigs.

For the HCA of *A. canelilla* twigs, it was also possible to analyze the formation of three distinct groups. Group I includes the months of August and October. Group II presents the months of September, February, and January. Furthermore, group III comprises the months of March, April, May, and June (see Figure 7).

**Figure 8:**
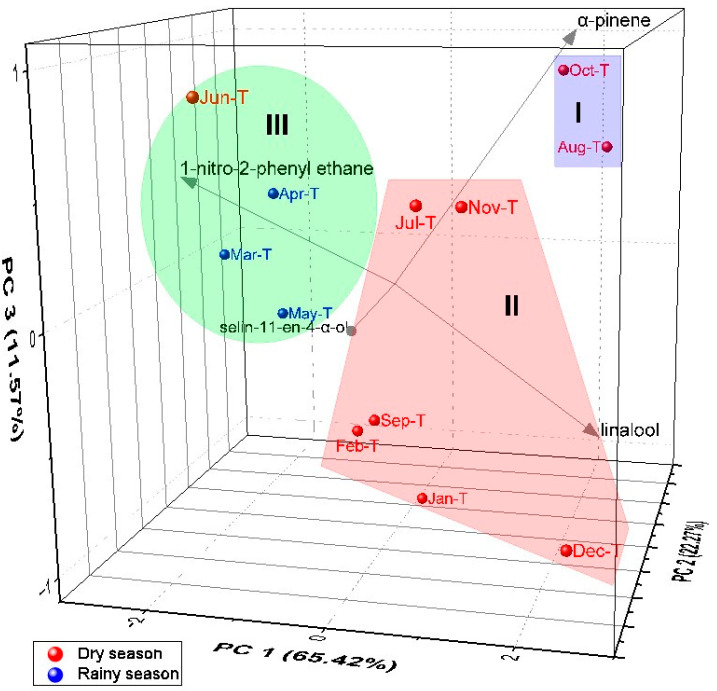
PCA analysis of the main compounds of essential oils from *A. canelilla* twigs.

Principal component analysis (PCA, Figure 8) elucidated 99.26% of the data variability. PC1 explained 65.42% of the variability and presented negative correlations with 1N2F (r = −2.45) and selin-11-en-4α-ol (r = −1.29), and presented positive correlations with α-pinene (r = 1.92) and linalool (r = 2.43). The second component (PC2) explained 22.27% of the variability, showing negative correlations with linalool (r = −0.06) and 1N2F (r = −0.28), and showing positive correlations with α-pinene (r = 0.79) and selin-11-en-4α-ol (r = 1.59). The third component (PC3) explained 11.57% of the data and showed negative correlations with linalool (r = −0.61) and selin-11-en-4α-ol (r = −0.46), and positive correlations with α-pinene (r = 1.05) and 1N2F (r = 0.46). In relation to the HCA, PCA analysis confirmed the formation of three distinct groups.

PCA and HCA analysis of *Aniba canelilla* leaves and twigs did not differentiate oil samples during the dry and rainy seasons. A previous study on the seasonality of essential oils from *Psidium friedrichsthalianum* leaves from Brazil did not show a separation of samples in the dry and rainy seasons [32]. Some species present variation in the concentrations of their constituents, but cannot be separated in chemometric analyses due to their metabolism not correlating with biotic, abiotic factors, and climatic parameters, which can interfere with metabolic pathways [33]. However, correlations were observed between climatic parameters and oil constituents and their compound classes, as mentioned previously (see Table 1).

## 3. Material and Methods

### 3.1. Plant Material and Climatic Data

The leaves and twigs of *A. canelilla* were collected from a specimen from the city of Belém, Pará state, Brazil (coordinates: 1°27′20.3″ S/48°26′18.1″ W). For this seasonal study, leaves (200 g) and twigs (120 g) were sampled on the 10th day of each month at 10 a.m. from August 2021 to July 2022. The specimen was collected in accordance with the Brazilian legislation relating to the protection of biodiversity (Sisgen A704928).

The climatic parameters (insolation, temperature, and rainfall) of the mentioned area were obtained monthly from the website of the National Institute of Meteorology (INMET, http://www.inmet.gov.br/portal/, accessed on 31 August 2022, from the Brazilian Government [34]).

### 3.2. Extraction and Oil Composition

The leaves and twigs were dried in a refrigerated room, ground, and subjected to hydrodistillation (in duplicate) using a Clevenger-type apparatus (3 h) according to the methodology described by Figueiredo et al. [35].

The chemical compositions of the obtained essential oils were analyzed with gas chromatography–flame ionization detector (GC-FID, Shimadzu Corporation, Tokyo, Japan) and gas chromatography–mass spectrometry (GC/MS, Shimadzu Corporation, Tokyo, Japan) simultaneously [35].

The individual components were identified by comparing their retention indices and mass spectra (molecular mass and fragmentation pattern) with the libraries of the GCMS-Solution system [26,27]. The retention index was calculated for all volatile components using a homologous series of C_8_-C_40_ *n*-alkanes (Sigma-Aldrich, Milwaukee, WI, USA) according to the linear equation of van Den Dool and Kratz [36]. GC-FID and GC-MS analyses were performed in duplicate.

### 3.3. Antioxidant Capacity

#### DPPH Radical Scavenging Method

The antioxidant capacity of the oils from seasonal samples was evaluated with the DPPH radical scavenging method [37,38]. Each essential oil sample from this seasonal study (5.0 µL, 10 mg/mL) was mixed with Tween 20 solution (0.5%, 50 µL, *w*/*w*) and then added to DPPH (0.5 mM, 1 mL) in ethanol. The absorbance was measured in a spectrophotometer (Ultrospec^TM^ 7000, Biochrom US, Holliston, MA, USA) at the beginning of the reaction, every 5 min during the first 30 min, and then at 30 min intervals until constant absorbance values were observed (reaction plateau, 2 h). Standard curves were prepared using Trolox (6-hydroxy-2,5,7,8-tetramethylchroman-2-carboxylic acid; Sigma-Aldrich, St. Louis, MO, USA), at concentrations of 30, 60, 150, 200, and 250 µg/mL and the same reaction mixture. The DPPH inhibition percentage calculated the radical scavenging activity of each sample according to the following equation, inhibition = 100 [(A − B)/A], where A and B are the blank and sample absorbance values in the end reaction. The results were expressed in milligrams of Trolox equivalents (mgTE/g) per gram of each sample. The total antioxidant activity was expressed as milligrams of Trolox, calculated utilizing the following equation, TE(mg/)g = [(A − B)/(A − C)] × [25/1000] × [250.29/1000] × [1000/10] × D, where A, B, and C are the blank, sample, and Trolox absorbance values in the end reaction, respectively, and D is the dilution factor. All experiments were triplicated.

### 3.4. Statistical Analysis

Statistical analysis was performed according to Santos et al. [23]. Statistical significance was assessed using the Tukey test (*p* < 0.05). GraphPad Prism software, version 8.0, was used to calculate Pearson’s correlation coefficients (r). Principal component analysis (PCA) was applied to verify the inter-relationship in the oil components (>2%). Hierarchical cluster analysis (HCA), considering Euclidean distance and complete linkage, was used to verify the similarity of oil samples based on the distribution of constituents selected in the previous PCA analysis.

## 4. Conclusions

The leaves showed higher essential oil yields than the twigs during this study. However, the yields showed no statistical difference between dry and rainy periods, indicating that the essential oil from the specimen can be extracted throughout the year.

The major constituent identified throughout the seasonal period in the essential oils from the leaves and twigs of *Aniba canelilla* was 1N2F. The results suggest that separating the leaves from the twigs is unnecessary, considering that 1N2F is present in all parts of the plant.

Methyleugenol was not identified in any of the study months—a fact described for the first time—which makes the specimen a reliable source of 1N2F. Furthermore, the oils from the twigs showed greater antioxidant capacity than those from the leaves. Therefore, this work contributes to the knowledge of the pharmacological potential of the species and encourages possible phytotherapeutic applications with the essential oils from the leaves and twigs of *A. canelilla*.

## Figures and Tables

**Table 1:** Correlation between yields, 1-nitro-2-phenylethane, main constituents, classes, and climatic parameters.

Yield/ Components	Temperature		Insolation		Precipitation	
	L	T	L	T	L	T
Oil yield	0.01	−0.11	0.30	−0.20	−0.17	0.10
1-nitro-2-phenylethane	−0.59 *	−0.47	−0.16	−0.37	0.61 *	0.60 *
Linalool	0.49	0.56	0.12	0.38	−0.18	−0.65 *
β-longipinene	0.55	0.26	0.68 *	−0.14	−0.64 *	−0.23
*E*-caryophyllene	0.43	0.06	0.16	−0.10	0.29	−0.33
Selin-11-en-α-ol	−0.24	−0.68 *	−0.13	−0.55 *	−0.37	0.72 *
Caryophyllene oxide	−0.11	−0.01	−0.37	−0.14	0.29	−0.26
α-pinene	0.29	0.65 *	0.24	0.67 *	−0.33	−0.42
Monoterpene hydrocarbons	0.35	0.69 *	0.28	0.71 *	−0.40	−0.51
Oxygenated monoterpenes	0.78 *	0.54	0.41	0.33	−0.60 *	−0.63 *
Sesquiterpene hydrocarbons	0.70 *	−0.08	0.49	−0.13	−0.70 *	−0.22
Oxygenated sesquiterpenes	−0.13	−0.70 *	−0.36	−0.43	0.41	0.48
Benzenoids	−0.58 *	−0.48	−0.14	−0.38	0.58 *	0.61 *

* Significant correlation (*p* < 0.05); L: leaves; T: twigs.

**Table 2:** Chemical composition of *Aniba canelilla* essential oils in this seasonal study.

RIC	RIL		August		September		October		November		December		January		February		March		April		May		June		July		Class
		*Aniba canelilla*	L	T	L	T	L	T	L	T	L	T	L	T	L	T	L	T	L	T	L	T	L	T	L	T	
		Oil Yields (%)	1.4	0.7	1.2	1.2	1.2	0.7	1.3	0.9	1.2	0.9	1.3	0.8	1.1	0.9	1.2	0.7	1.2	1.0	1.6	0.7	1.2	0.4	1.7	0.8	
		Oil Constituents (%)	(%)																								
933	932 ^a^	α-pinene	0.3	2.2	0.1	0.2	0.8	2.2	0.3	1.0	0.8	0.6	0.3	0.2	0.6	0.1		tr	0.2	0.4	tr	0.1	2.2	0.1	tr	0.8	MH
948	946 ^a^	camphene	tr	0.1			tr	0.1		tr	tr	tr	tr		tr								0.1			tr	MH
958	952 ^a^	benzaldehyde	1.1	0.1	0.4	0.1	0.5	0.1	0.5	tr	1.2	0.1	0.7		0.8		1.0		0.7		0.5	tr	0.5		0.4	0.1	BZ
973	974 ^a^	sabinene									tr		0.1	0.1	0.1	tr	0.6		tr				0.4	0.6	0.7	0.4	MH
977	974 ^a^	β-pinene	0.3	1.2	0.1	0.2	0.8	1.2	0.2	0.7	0.6	0.5	0.3	0.2	0.6	0.1	0.1	tr	0.2	0.3	tr	0.1	1.3	0.2	0.2	0.7	MH
984	983 ^b^	benzoic acid nitrile		tr			0.2		0.2		0.2	tr	0.2		0.2		0.1				0.1		0.5		0.2		BZ
991	988 ^a^	myrcene		0.3			tr	0.3		0.1		0.1		0.1	tr	tr				0.1		tr				0.1	MH
1006	1002 ^a^	α-phellandrene		0.1				0.1		0.1		tr				tr										tr	MH
1011	1008 ^a^	δ-3-carene		0.2				0.2		0.1		0.1		tr	tr					tr			tr			0.1	MH
1024	1020 ^a^	*p*-cymene	0.1	0.3	tr	tr	0.1	0.3	tr	0.1	0.1	0.1	tr		0.1	0.1				0.1		tr	0.2	tr		0.1	MH
1028	1024 ^a^	limonene	0.1		tr		0.4		0.1		0.2		0.1		0.2	0.2			tr				0.5		tr		MH
1029	1025 ^a^	β-phellandrene		1.1		0.2		1.1		0.6		0.4								0.3		0.1				0.6	MH
1031	1026 ^a^	1.8-cineole	0.2	0.3	0.1	0.1	0.2	0.2	0.1	0.1		0.1			0.2		tr			0.1		tr	0.3		tr	0.1	OM
1036	1032 ^a^	*Z*-β-ocimene		0.2				0.2		0.1		0.1		0.2						0.1				0.1		0.1	MH
1041	1036 ^a^	benzene acetaldehyde	1.0	0.1	1.1		1.3	0.1	1.4	0.1	0.3	0.2	1.2	0.1	1.1	0.1	0.6		0.6		1.7	tr	0.7		1.8	0.1	BZ
1046	1044 ^a^	*E*-β-ocimene		0.3		0.1		0.3		0.2		0.2		0.1		0.1						tr		tr		0.2	MH
1058	1054 ^a^	γ-terpinene		tr				tr									tr						tr	tr		tr	MH
1069	1059 ^a^	acetophenone	0.1								0.1		0.1		tr		0.1										BZ
1071	1067 ^a^	*cis*-linalool oxide	0.1	0.1	tr		tr	tr	tr	tr		0.1		tr	tr	0.1	0.1					tr				tr	OM
1088	1084 ^a^	*trans*-linalool oxide	0.1	0.1	tr				tr	0.1	0.1	0.1	0.1	0.1	0.1	0.1	0.1					tr	0.1				OM
1089	1086 ^a^	terpinolene						0.1																			MH
1100	1095 ^a^	linalool	2.2	16.1	2.7	13.0	3.5	14.2	3.1	13.2	3.4	20.1	2.3	12.6	2.3	12.0	2.5	4.5	2.6	6.1	1.9	6.5	2.6	5.5	2.7	12.1	**OM**
1137	1134 ^a^	benzeneacetonitrile	0.3	0.2	0.3		0.1	0.2	0.2	0.2	0.3	0.2	0.2	0.1	0.2	0.2	0.1	0.1	0.1	0.1	0.2	0.2	0.3	0.2	0.3	0.1	BZ
1139	1135 ^a^	*trans*-pinocarveol					0.1		0.1						0.1		0.1				0.1						OM
1177	1174 ^a^	terpinen-4-ol	tr	0.1	tr		tr	0.1	0.1	0.1	0.1	0.1	tr	0.1	tr	0.1						0.1	tr			tr	MH
1190	1186 ^a^	α-terpineol	0.3	0.8	0.4	0.8	0.5	0.6	0.4	0.7	0.5	1.0	0.3	0.8	0.3	0.8	0.3	0.6	0.3	0.5	0.3	0.7	0.4	0.4	0.4	0.5	OM
1195	1195 ^a^	myrtenal	0.1		0.1		0.1		0.1				0.1		0.1		tr						0.1				OM
1228	1227 ^a^	nerol		tr						tr		0.1		0.1	tr	0.1						0.1					OM
1255	1249 ^a^	geraniol		0.2	tr	0.1	0.1	0.1	tr	0.2	tr	0.3	tr	0.3	0.1	0.3		0.2		0.2		0.3				tr	OM
1256	1254 ^a^	2-phenylethyl acetate	tr	0.1	0.1			0.1		0.1	0.1	0.1	tr	0.1	tr			0.1				0.1		0.1	0.1	0.1	BZ
1308	1294 ^a^	1-nitro-2-phenylethane	80.4	71.9	80.2	81.7	71.9	75.2	75.3	77.7	74.6	71.3	81.2	75.6	68.0	82.0	85.2	89.9	85.0	87.9	83.1	85.6	76.2	89.8	83.7	79.8	**BZ**
1351	1345 ^a^	α-cubebene			0.1		0.1		tr						0.1												SH
1357	1356 ^a^	eugenol	0.2	0.6	0.5	0.7	0.3	0.7	0.3	0.6	0.3	0.6	0.3	0.6	0.4	0.8	0.2	0.9	0.2	0.9	0.3	1.0	0.1	0.8	0.2	0.5	BZ
1377	1374 ^a^	α-copaene	0.5		0.7		1.2		1.0		0.4		0.2		1.6		0.2		0.3		0.4		0.6		0.8		SH
1393	1389 ^a^	β-elemene	tr		tr		0.1		0.1				tr		0.1							tr	tr				SM
1408	1400 ^a^	β-longipinene	1.9		0.6		2.5		1.0		1.5	0.1	0.6				0.7		0.6				2.4		4.8		**SM**
1420	1417 ^a^	*E*-caryophyllene	0.5	0.2	4.9	0.2	5.4	0.2	5.1	0.3	1.0	0.3	0.6	0.3	6.6	0.2	0.2	0.2	1.0	0.2	1.9	0.2	0.8	0.1	1.4	0.3	SM
1441	1439 ^a^	2-phenylethyl butanoate	0.1								0.1				0.1								0.1				BZ
1454	1452 ^a^	α-humulene	0.1	tr	0.5		0.7	tr	0.6	tr	0.3	tr	0.1	tr	0.6		tr		0.1		0.2	tr	0.3		0.5	tr	SH
1487	1490 ^a^	2-phenylethyl 3-methylbutanoate		0.1						0.1		0.1	0.3	0.2			0.2						0.4	0.1	0.2	0.2	BZ
1496	1498 ^a^	α-selinene	0.1	0.1	0.1		0.2		0.1	0.1	0.1	0.1	0.1	0.1	0.2			0.1				0.1	0.1	tr	0.1	0.1	SH
1509	1505 ^a^	β-bisabolene	0.1		0.1		0.2		0.1		0.1		0.1		0.2							tr	0.1		0.1		SH
1524	1521 ^a^	*trans*-calamenene	0.1										0.1		0.2		tr				0.1						SH
1525	1522 ^a^	δ-cadinene		tr	0.1		0.2		0.2			tr		0.1								0.1			0.1	tr	SH
1564	1561 ^a^	*E*-nerolidol		0.1				0.1		0.1	tr	0.1	tr	0.1	tr	0.1		0.1		0.1		0.1		tr		0.1	OS
1571	1565 ^a^	3*Z*-hexenyl benzoate			0.1						0.1		0.1		0.1												BZ
1578	1577 ^a^	spathulenol	0.1	tr				0.1	tr	0.1	0.1	0.1	tr	0.1		0.1		0.1		0.1		0.2	0.1	0.1		0.1	OS
1584	1582 ^a^	caryophyllene oxide	5.1	0.2	3.4		4.9	0.2	4.9	0.3	5.2	0.3	4.5	0.3	5.6	0.2	4.8	0.1	5.4	0.2	5.5	0.4	4.2	0.2	0.7	0.3	OS
1588	1590 ^a^	β-copaen-4α-ol	tr		tr		tr		tr				tr		0.1								0.1				OS
1599	1600 ^a^	guaiol		tr				0.1		0.1		0.1		0.1		0.1		tr		0.1		0.1		tr		0.1	OS
1610	1608 ^a^	humulene epoxide II	0.4		0.2		0.2		0.3		0.3		0.3		0.4		0.3		0.2		0.3	tr	0.2		tr		OS
1630	1627 ^a^	1-*epi*-cubenol	0.1	tr	tr		0.1		0.1	tr		tr		0.1	0.1	tr						0.1	0.1				OS
1634	1639 ^a^	caryophylla-4(12),8(13)-dien-5α-ol	0.3		0.2		0.3		0.3								0.8		0.2			tr					OS
1637	1639 ^a^	caryophylla-4(12),8(13)-dien-5β-ol	0.8		0.6		0.8		1.0		1.3		1.0		1.7		0.2		0.9		1.4		0.9				OS
1656	1651 ^a^	pogostol							1.0								1.0						1.0	1.9	0.6	1.8	OS
1656	1658 ^a^	selin-11-en-4α-ol	0.9	1.5	0.8	1.3	0.9	1.5					0.6	1.9	1.1	1.6		2.5	0.9	2.0	1.0	2.4					OS
1659	1661 ^a^	*allo*-himachalol																			0.2		0.7				OS
1672	1668 ^a^	14-hydroxy-9-*epi*-*E-*caryophyllene	0.5		0.3										tr		0.4						0.4				OS
1669	1670 ^a^	bulnesol		tr				tr		0.1		0.1		0.1		0.1						0.1				0.1	OS
1678	1676 ^a^	mustakone	0.1		tr								tr		0.1												OS
1759	1759 ^a^	cyclocolorenone		0.4				0.2		0.4		0.1		0.8				0.5		0.2		0.5				0.1	OS
Monoterpene hydrocarbons			0.7	5.9	0.3	0.6	2.1	5.9	0.6	3.0	1.7	2.1	0.7	0.9	1.6	0.6	0.7	0.1	0.5	1.4	0.1	0.2	4.8	1.0	1.0	3.2	
Oxygenated monoterpenes			2.9	17.6	3.3	14.0	4.5	15.2	3.9	14.4	4.0	21.8	2.8	14.0	3.3	13.5	3.0	5.3	2.9	6.8	2.3	7.7	3.5	5.8	3.2	12.8	
Sesquiterpene hydrocarbons			3.2	0.3	7.2	0.2	10.5	0.2	8.2	0.4	3.4	0.5	1.7	0.5	9.5	0.2	1.1	0.3	1.9	0.2	2.6	0.4	4.2	0.1	7.8	0.5	
Oxygenated sesquiterpenes			8.2	2.3	5.5	1.3	7.1	2.1	7.6	1.0	6.9	0.9	6.4	3.4	9.1	2.2	7.4	3.3	7.6	2.7	8.3	3.9	7.5	2.2	1.4	2.6	
Benzenoids			83.1	73.1	82.5	82.4	74.4	76.3	77.9	78.8	77.0	72.5	84.1	76.7	70.8	83.1	87.5	91.0	86.6	88.9	85.8	87.0	78.8	90.8	86.8	80.8	
Total			98.0	99.1	98.8	98.4	98.5	99.7	98.2	97.6	93.0	97.8	95.8	95.5	94.3	99.6	99.7	99.8	99.4	99.9	99.0	99.2	98.8	99.9	100.0	99.8	

RIC = calculated retention index (Rtx-5ms column); RIL = literature retention index; ^a^ = Adams, 2007 [26]; ^b^ = Mondello, 2011 [27]; tr: traces (<0.1%); main constituents in bold, *n* = 2 (standard deviation was less than 2.0); MH = monoterpene hydrocarbons; OM = oxygenated monoterpenes; SH = sesquiterpene hydrocarbons; OS: oxygenated sesquiterpenes; BZ: benzenoids.

## Data Availability

Data are contained within the article.

## Appendix A

**Table A1:** Chemical constituents of *Aniba canelilla* essential oils in this seasonal study.

RIC	RIL	Oil Constituents (%)	RT
933	932 ^a^	α-pinene	5.820
948	946 ^a^	camphene	6.225
958	952 ^a^	benzaldehyde	6.485
973	974 ^a^	sabinene	6.900
977	974 ^a^	β-pinene	7.020
984	983 ^b^	benzoic acid nitrile	7.180
991	988 ^a^	myrcene	7.375
1006	1002 ^a^	α-phellandrene	7.848
1011	1008 ^a^	δ-3-carene	8.035
1024	1020 ^a^	*p*-cymene	8.495
1028	1024 ^a^	limonene	8.650
1029	1025 ^a^	β-phellandrene	8.669
1031	1026 ^a^	1.8-cineole	8.740
1036	1032 ^a^	*Z*-β-ocimene	8.933
1041	1036 ^a^	benzene acetaldehyde	9.125
1046	1044 ^a^	*E*-β-ocimene	9.309
1058	1054 ^a^	γ-terpinene	9.723
1069	1059 ^a^	acetophenone	10.145
1071	1067 ^a^	*cis*-linalool oxide	10.210
1088	1084 ^a^	*trans*-linalool oxide	10.805
1089	1086 ^a^	terpinolene	10.827
1100	1095 ^a^	linalool	11.240
1137	1134 ^a^	benzeneacetonitrile	12.770
1139	1135 ^a^	*trans*-pinocarveol	12.820
1177	1174 ^a^	terpinen-4-ol	14.435
1190	1186 ^a^	α-terpineol	14.990
1195	1195 ^a^	myrtenal	15.235
1228	1227 ^a^	nerol	16.565
1255	1249 ^a^	geraniol	17.690
1256	1254 ^a^	2-phenylethyl acetate	17.795
1308	1294 ^a^	1-nitro-2-phenylethane	19.803
1351	1345 ^a^	α-cubebene	21.810
1357	1356 ^a^	eugenol	22.110
1377	1374 ^a^	α-copaene	22.930
1393	1389 ^a^	β-elemene	23.605
1408	1400 ^a^	β-longipinene	24.230
1420	1417 ^a^	*E*-caryophyllene	24.728
1441	1439 ^a^	2-phenylethyl butanoate	25.565
1454	1452 ^a^	α-humulene	26.096
1487	1490 ^a^	2-phenylethyl 3-methylbutanoate	27.425
1496	1498 ^a^	α-selinene	27.815
1509	1505 ^a^	β-bisabolene	28.340
1524	1521 ^a^	*trans*-calamenene	28.920
1525	1522 ^a^	δ-cadinene	28.902
1564	1561 ^a^	*E*-nerolidol	30.455
1571	1565 ^a^	3*Z*-hexenyl benzoate	30.710
1578	1577 ^a^	spathulenol	31.045
1584	1582 ^a^	caryophyllene oxide	31.203
1588	1590 ^a^	β-copaen-4α-ol	31.395
1599	1600 ^a^	guaiol	31.815
1610	1608 ^a^	humulene epoxide II	32.235
1630	1627 ^a^	1-*epi*-cubenol	32.970
1634	1639 ^a^	caryophylla-4(12),8(13)-dien-5α-ol	33.090
1637	1639 ^a^	caryophylla-4(12),8(13)-dien-5β-ol	33.230
1656	1651 ^a^	pogostol	33.893
1656	1658 ^a^	selin-11-en-4α-ol	33.930
1659	1661 ^a^	*allo*-himachalol	34.025
1672	1668 ^a^	14-hydroxy-9-*epi*-*E-*caryophyllene	34.540
1669	1670 ^a^	bulnesol	34.390
1678	1676 ^a^	mustakone	34.770
1759	1759 ^a^	cyclocolorenone	37.640

RIC = calculated retention index (Rtx-5ms column); RIL = literature retention index; ^a^ = Adams, 2007 [26]; ^b^ = Mondello, 2011 [27]; RT: retention time.

**Table A2:** DPPH radical scavenging of the monthly oils of *Aniba canelilla*.

Sample	Leaves		Twigs	
	Inhibition (%) *	TEAC (mg.TE/g) *	Inhibition (%) *	TEAC (mg.TE/g) *
August	17.9 ± 1.3 ^a,d,e^	11.8 ± 7.4	40.2 ± 1.3 ^a,e,g^	226.8 ± 7.2
September	31.3 ± 0.5 ^b^	174.3 ± 3.0	42.0 ± 3.9 ^a,b^	236.6 ± 22.2
October	24.7 ± 1.3 ^c^	138.6 ± 7.3	40.2 ± 1.3 ^a,b,e,g^	226.8 ± 7.2
November	19.8 ± 1.0 ^d^	110.8 ± 6.0	33.0 ± 0.9 ^c,h^	186.2 ± 5.2
December	15.6 ± 0.5 ^e,f^	87.0 ± 3.2	30.2 ± 1.2 ^c,d^	170.4 ± 6.8
January	22.5 ± 0.6 ^c^	126.2 ± 3.6	34.4 ± 1.1 ^c,d,h^	193.9 ± 6.1
February	23.3 ± 0.8 ^c^	130.3 ± 4.9	39.8 ± 1.5 ^a,b,e,g^	224.5 ± 8.4
March	24.3 ± 1.1 ^c^	136.3 ± 6.2	40.6 ± 1.0 ^a,b,e,g^	229.2 ± 6.0
April	18.8 ± 0.4 ^a,d^	105.3 ± 2.3	37.2 ± 0.4 ^a,b,h^	210.1 ± 2.3
May	16.4 ± 1.0 ^a,e^	91.9 ± 5.8	36.7 ± 2.2 ^e,h^	206.8 ± 12.3
June	13.4 ± 0.3 ^f^	74.9 ± 2.0	20.8 ± 1.0 ^f^	117.6 ± 5.4
July	17.2 ± 0.3 ^a,d,e^	96.6 ± 1.6	36.8 ± 0.9 ^g,h^	207.7 ± 4.9

* Values are expressed as means ± standard deviations (*n* = 3). Values with the same letters in the column do not differ statistically in the Tukey test (*p* > 0.05).
